# Formulation of Cinnamon (*Cinnamomum verum*) oil loaded solid lipid nanoparticles and evaluation of its antibacterial activity against Multi-drug Resistant *Escherichia coli*

**DOI:** 10.1186/s12906-022-03775-y

**Published:** 2022-11-09

**Authors:** Mehran Nemattalab, Masoumeh Rohani, Mehdi Evazalipour, Zahra Hesari

**Affiliations:** 1 Department of Pharmaceutics, School of Pharmacy, Guilan University of Medical Sciences, Rasht, Iran; 2 Department of Microbiology, School of Medicine, Guilan University of Medical Sciences, Rasht, Iran; 3 Student Research Committee, School of Pharmacy, Guilan University of Medical Sciences, Rasht, Iran; 4 Department of Pharmaceutical Biotechnology, School of Pharmacy, Guilan University of Medical Sciences, Rasht, Iran

**Keywords:** *Escherichia coli*, Cinnamon Oil, Solid lipid nanoparticles, Minimum inhibitory concentration, Anti-biofilm activity

## Abstract

Today, the increment in microbial resistance has guided the researches focus into new antimicrobial compounds or transmission systems. *Escherichia coli* (*E. coli*) is an opportunistic pathogen, producing a biofilm responsible for a wide range of nosocomial infections which are often difficult to eradicate with available antibiotics. On the other hand, *Cinnamomum verum* (cinnamon oil) (CO) is widely used as a natural antibacterial agent and Solid lipid nanoparticles (SLNs) are promising carriers for antibacterial compounds due to their lipophilic nature and ease of transmission through the bacterial cell wall. In this study, nanoparticles containing cinnamon oil (CO-SLN) were prepared by dual emulsion method and evaluated in terms of particle size, shape, entrapment efficiency (EE), transmission electron microscopy (TEM), oil release kinetics, and cell compatibility. The antibacterial activity of CO-SLN and CO against 10 drug-resistant *E. coli* strains was investigated. The anti-biofilm activity of CO-SLN on the selected pathogen was also investigated. Nanoparticles with an average size of 337.6 nm, and zeta potential of -26.6 mV were fabricated and their round shape was confirmed by TEM images. The antibacterial effects of CO-SLN and CO were reported with MIC Value of 60–75 µg/mL and 155–165 µg/mL and MBC value of 220–235 µg/ml and 540–560 µg/ml, respectively. On the other hand, CO-SLN with 1/2 MIC concentration had the greatest inhibition of biofilm formation in 24 h of incubation (55.25%). The data presented indicate that the MIC of CO-SLN has significantly reduced and it seems that SLN has facilitated and promoted CO transmission through the cell membrane.

**Supplementary Information:**

The online version contains supplementary material available at 10.1186/s12906-022-03775-y.

## Introduction

*Escherichia coli* (*E. coli*) is a gram-negative, rod-shaped bacterium of the Enterobacteriaceae family [1]. Most strains are harmless, but a small number of strains can cause various intestinal and extraintestinal infections, including diverse intra-abdominal, urinary tract infections (UTIs), pulmonary, skin, soft tissue infections, diarrhea, bacteremia, and newborn meningitis (NBM) through mechanisms such as attachment, escape of the host immune defense, and production of toxins [2–4]. This microorganism can form biofilms, which play an effective role in causing infection and resistance to eradication [5, 6]. *E. coli* is an opportunistic pathogen and its biofilm is responsible for a wide range of nosocomial infections that are often difficult to eradicate with antibiotics, hence, continuous administration of antibiotics leads to antibiotic resistance. The emergence of multidrug resistance in *E. coli* is a serious threat to global health, which the World Health Organization (WHO) has promulgated antimicrobial resistance as one of the major universal threats in the 21st century [7–10].

*Cinnamomum verum* (CO) derived from the Cinnamomum plants of the Lauraceae family has been used for centuries in traditional medicine as a natural antibacterial agent and its effectiveness against Gram-positive and Gram-negative bacteria has been proven in a way that effectively prevent the bacterial growth by morphological destruction of the bacteria [11–14]. There are numerous reports of various functions of cinnamon such as antioxidant, anti-cholesterol, antimicrobial and antifungal, anti-diabetic, anti-inflammatory, relieving stomach pain, treating diarrhea and gastrointestinal upset [15]. In the food industry, CO is used as a flavor and aroma additive with broad-spectrum activity against pathogenic microorganisms in food. Unfortunately, the current use of CO is limited due to its volatile chemical instability in the vicinity of air, light, humidity, and high temperatures [16].

Nowadays, the effectiveness of a drug substance depends not only on the properties of the drug but also on the carrier system, which may lead to the controlled and localized release of the active substance based on specific clinical goals [17]. Solid lipid nanoparticles (SLNs) have been recommended as a new drug delivery carrier, mainly for lipophilic active ingredients [18] that can physically protect sensitive drugs (oxidation, light, humidity) and provide a controlled drug release, high surface area to volume ratio, and high drug loading capacity [19, 20]. They are composed of solid biodegradable lipids in aqueous colloidal dispersions, unifying the benefits of liposomes and fat emulsions simultaneously [21]. SLNs are composed of a high melting point triglyceride as the solid/liquid core and a phospholipid coating with low systemic toxicity and also low cytotoxicity [22]. Basically, biocompatible well-tolerated lipids can be used in the composition of SLNs including triglycerides, cholesterol, etc. High-pressure homogenization is a cost-effective and relatively simple method for large-scale SLN production [23].

In this study, CO-SLNs were prepared in order to improve its physicochemical and antimicrobial properties. Several investigations have confirmed the positive influence of lipid carriers on increasing the antimicrobial effectiveness of herbal oils [24–26]. Also, various cinnamon oil loaded nano delivery systems have been developed including Nanostructured lipid carriers (NLC) [27], alginate-calcium nano/micro particle [28], chitosan nanoparticles [29], etc.). Yet, to the best of our knowledge, there was no study on probable influences of SLN as a lipidic carrier on antibacterial properties of cinnamon oil, using a high-energy method (ultrasonic) with a non-ionic surfactant (Tween 80) and co-surfactant (lecithin). The CO-SLN antibacterial activity against *E. coli* ATCC 25,922 and multidrug-resistant organisms isolated from the hospital was evaluated in comparison with CO. In addition, the anti-biofilm activity of nanoparticles on selected pathogen was investigated using the crystal violet method.

## Methods

### Preparation of CO loaded SLNs

In this study, an emulsification ultrasonic-homogenization method was used to prepare solid lipid nanoparticles containing cinnamon oil [30, 31]. Briefly, 1 ml CO (was bought commercially and analyzed by GC-MS to confirm its quality (supplementary data 1)) was dissolved in 10 ml methanol (Merck, Darmstadt, Germany), adding 800 µl tween 80 (Merck, Darmstadt, Germany). 200 mg Lecithin (Merck) & 100 mg Cholesterol (Sigma-Aldrich) were dissolved in 10 ml dichloromethane (Merck, Darmstadt, Germany). Methanolic and dichloromethane solutions were mixed manually. The resultant primary organic phase was mixed with 10 ml of PVA 4% w/v (Merck, Darmstadt, Germany) solution and homogenized for 10 min at 15,000 rpm using an ultrasound probe sonicator (Hielscher UP400s, Germany) to produce a white cloudy emulsion. The resultant o/w was subjected to a Rota evaporator at 45 °C for complete evaporation of the organic phase.

### Characterization of CO-SLN

#### Particle size, poly dispersity index (PDI) measurement and zeta potential

The particle size and zeta potential of nanoparticles was determined using a Zetasizer 1,033,439 (Malvern Instrument, UK). 15 µl of the sample was suspended in 1 ml double-distilled water and the average particle size was calculated by Zetasizer Ver. 6.01 software at 25 °C with a count rate of 206.3 kcps and measurement position of 4.65 mm. For evaluation of size distribution (monodisperse or polydisperse nature) of nanoparticles, the polydispersity index was determined. The higher polydispersity index values (≥ 0.7) indicate a high level of non-uniformity.

#### Transmission Electron Microscopy (TEM)

The shape and morphology of the nanoparticles was investigated, using a TEM microscope (Zeiss-EM10C-100 KV, Germany) operated at 80 kV. Suspension of the nanoparticles, was contrasted with uranyl acetate and placed on 200–300 mesh grids, coated with Formar (a low absorption resin). The grids were allowed to dry by evaporation for TEM analysis.

#### Fourier transform infrared (FTIR) spectrometry

Pure CO, CO-SLN, cholesterol, tween 80 and lecithin were subjected to FTIR spectrometry (PerkinElmer ES Version 10.5.3, USA) to examine the probable incompatibilities between CO and incorporated excipients. FTIR Spectra were collected at a resolution of 4 cm^− 1^and given as the ratio of 21 single beam scans to the same number of background scans in pure KBr.

#### Entrapment efficiency (EE)

500 mg of CO-SLN was dispersed in 10 mL of distilled water. The aqueous dispersion was centrifuged with 6000 rpm for 20 min at room temperature. The amount of free CO was detected in the supernatant fluid by UV spectroscopy (PerkinElmer, USA) at 270 nm [32] and the percent of entrapped oil was calculated using the following equation:

EE (%) = (W _initial CO_-W _free CO_)/ W _initial CO_×100

### *In-vitro* release and release kinetic study

1 g of nanoparticle was incorporated into the dialysis bag with a molecular weight cutoff of 14 kDa (Sigma, Steinheim, Germany) sealed in both sides. The bag was immersed into the 100 mL phosphate buffer pH 7.4 (Sigma-Aldrich) containing 2% v/v tween 80 as receptor medium, at 25 °C with constant stirring (100 rpm). Samples (2 ml) were withdrawn in the tubes at various time points of 1, 3, 6, 24, 48, and 72 h and substituted with fresh medium to maintain the sink condition. Furthermore, analyses of the content of CO were performed using UV–Visible spectrophotometry in 270 nm [33].

The in vitro release data was incorporated to investigate the release kinetics of CO-SLN using Zero order, First order, Higuchi, Korsmeyer-Peppas and Hixson crowell mathematical kinetic models [34].

### Cell compatibility assay

The antiproliferative activities of the CO-SLN on HU02 (Foreskin fibroblast) cell lines were evaluated using MTT assay. Briefly, cells were incubated in a 96-well plate with a density of 5×l0^3^ cells/cm^2^ in 100 µl of DMEM medium for 24 h under a humidified atmosphere with 5% CO_2_. The cells were treated with CO-SLN (25, 50 mg/ml) and CO (50 mg/ml) while all the sample media contained 2% v/v tween 80. The cells were incubated with MTT solution (Sigma; 5 mg/ml of PBS) for 3 h at 37 ºC, after 24 h of treatment. Subsequently, the medium was removed, and the precipitates formed were dissolved in 150 µl DMSO per well. Absorbance was recorded using a Biotek Epoch™ microplate reader at 570 nm (n = 3).

### Antibacterial assay

#### *E.coli* isolated strains and antimicrobial susceptibility test

In this study, the antibacterial activity of CO-SLN and CO against a standard sample of *E. coli* (ATCC 25,922) was assessed. In addition, 10 MDR *E. coli* isolates resistant to 8 antibiotics (*Ampicillin, Gentamicin, Amikacin, Ceftazidime, Cefepime, Ceftriaxone, Ciprofloxacin, Trimethoprim-Sulfamethoxazole*), obtained from the 17 Shahrivar Children’s Hospital (Rasht, Iran) and were subjected to CO-SLN and CO for further evaluation of antibacterial effects of the compounds.

#### MIC and MBC determination

The minimum inhibitory concentration (MIC) of CO-SLN and CO were investigated using micro broth dilution assay in a 96- well plate. Based on the preliminary study results, the CO-SLN (80 − 50 µg/ml) and CO (170 − 140 µg/ml) were serially diluted in sterile Muller-Hinton broth (Merck, Germany) in each well. The final concentration of *E. coli* was adjusted to 10^6^ CFU/mL for each well and incubated at 37 ° C for 20 h. The MIC values were determined as the lowest concentration of drugs that inhibited bacterial growth. To determine the Minimum Bactericide Concentration (MBC), 100 µL content of no growth wells were cultured in Muller-Hinton agar (Merck, Germany) and were incubated at 37 °C for 24 h. The MBCs were reported as the lowest concentration that resulted in killing 99.9% of bacterial cells.

#### Anti-biofilm assay

This assay was performed according to the method of Hou, et al. with small modifications [35]. The most resistant strain was used to inoculate the Muller-Hinton broth medium containing different concentrations of CO-SLN and CO (1/2 × MIC, 1/4 × MIC, 1/8 × MIC) incubated for 24 and 48 h at 37 ° C. The culture medium with bacteria was used as a positive control and the culture medium without bacteria and CO-SLN was used as a negative control. Next, the contents of the wells were removed and 200 µl of methanol was added to each well for 15 min to fix the biofilms. The present biofilms were stained with a 1% crystal violet (Sigma) solution for 10 min at room temperature ​and the stained biofilm cells were dissolved in 200 µl of 95% ethanol. The OD of samples was evaluated at 570 nm. The Anti-biofilm index was calculated using the following formula.

Inhibition / eradication % = (OD control-OD sample) /OD control × 100%

### Statistical analysis

Independent t-test and one-way ANOVA tests were performed, using SPSS 22.0 software. All experiments were screened in triplicate.

## Results

### Nanoparticle preparation and characterization

#### Particle size, zeta potential and TEM analysis

**Fig. 1:**
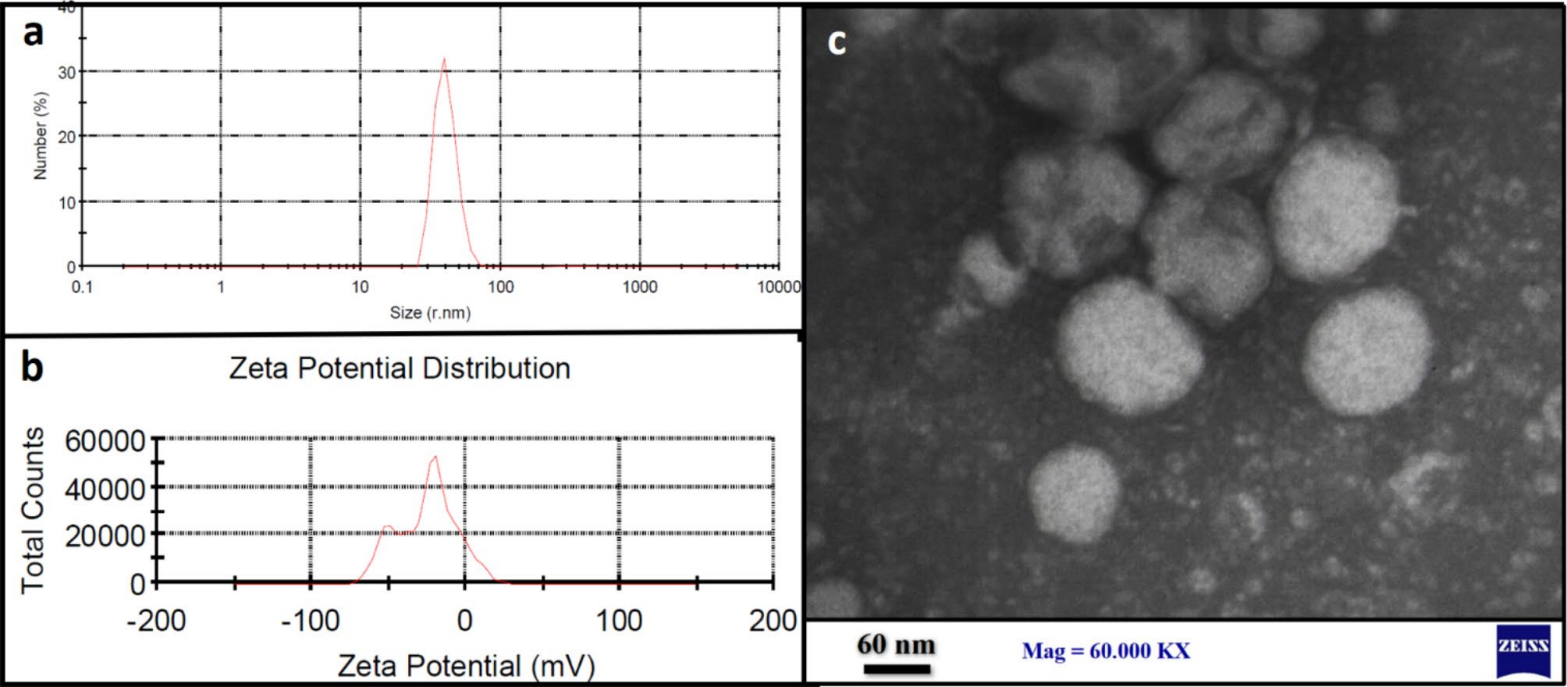
CO-SLN characterization; (a) particle size analysis by DLS. (b) zeta potential distribution; (c) TEM two-dimensional morphological description

The particle size of CO-SLN was reported with the average of 40.65 nm (shown in Fig. 1a). Also, the zeta potential of the nanoparticles was measured − 26.5 mV (Fig. 1b) that as a relatively high zeta potential results in stronger electrostatic repulsion which prevents particle aggregation and leads to better size stability. TEM provides two-dimensional morphological information, size, shape, and other general aspects. Figure 1c shows the TEM images of CO-SLN which reveals the uniform round shaped with clear edge particles.

#### FTIR spectrometry

**Fig. 2:**
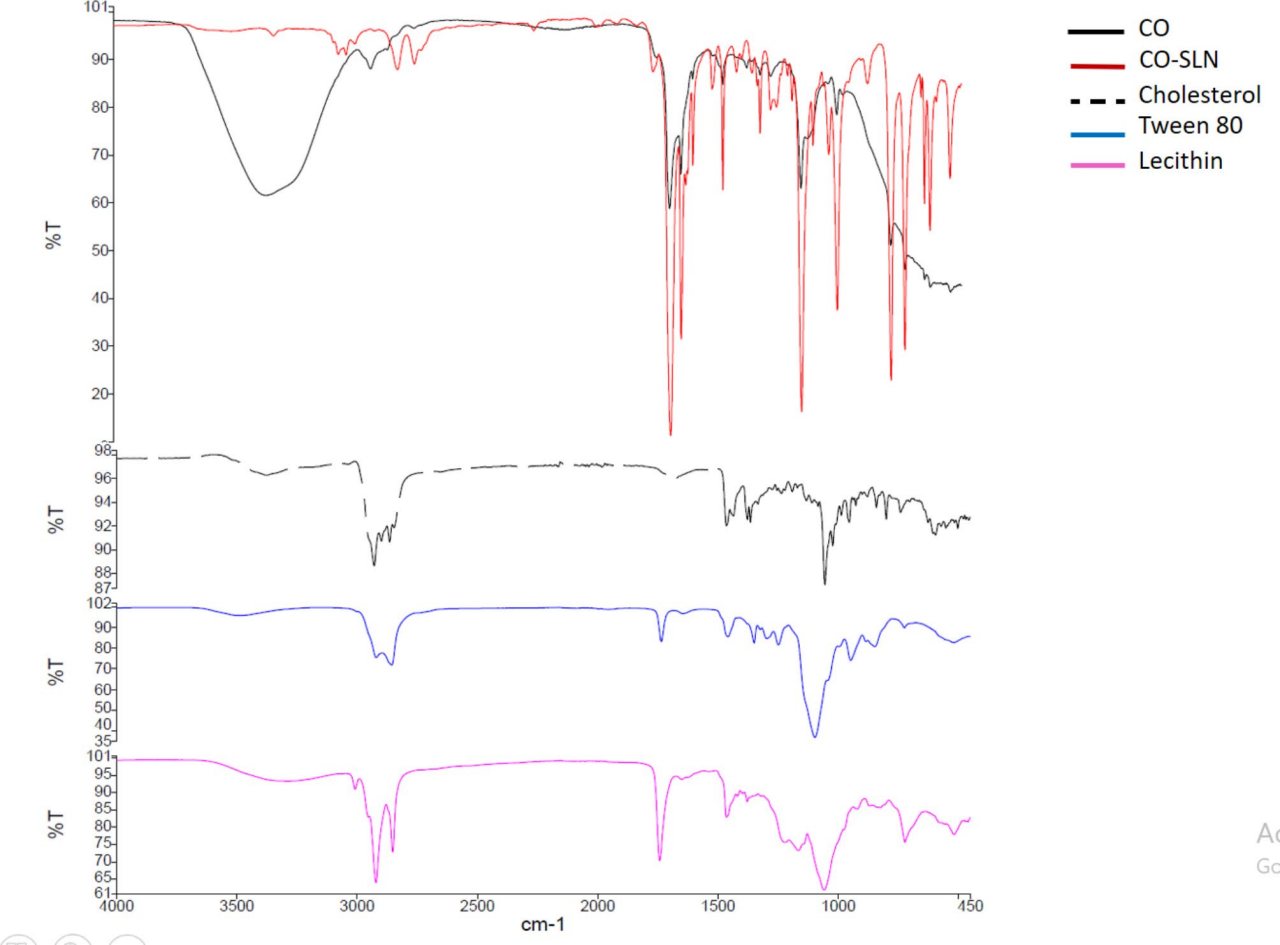
FTIR spectroscopy of pure CO (black), CO-SLN (red) (with common peaks around 1425 and 2800 cm^− 1^), cholesterol (dotted), tween 80 (blue) and lecithin (pink)

Figure 2 presents the FTIR spectra of CO and CO-SLN along with SLN constituents including cholesterol, lecithin and tween 80. The FTIR spectra of CO revealed the intense absorption peaks at 1425 cm^− 1^ (aromatic C–C in the ring). The peak at 1425 cm^− 1^ is related to the aromatic ring of cinnamaldehyde, the major compound in CO. Also, there are other peaks at 2829–2885 cm^− 1^ (C–H stretching or carboxyl OH stretching), 1685 cm^− 1^ (CO stretching of aldehyde), and 1641 cm^− 1^ (alkene CC stretching in 3 ring).

In the CO-SLN FTIR spectrum, the peak at the regions between, 1685–1769 cm^− 1^, and 1425–1513 cm^− 1^ are assigned to the presence of CO in CO-SLN spectrum, indicating the successful entrapment of CO in CO-SLN and confirming no chemical interaction between CEO and CH-NLC components.

#### % entrapment efficiency (EE)

The percentage of incorporated CO in the lipid matrix was evaluated. Incorporation of CO led to high entrapment efficiency (79.1%), probably due to its lipophilic character.

### In vitro release and release kinetic studies

**Fig. 3:**
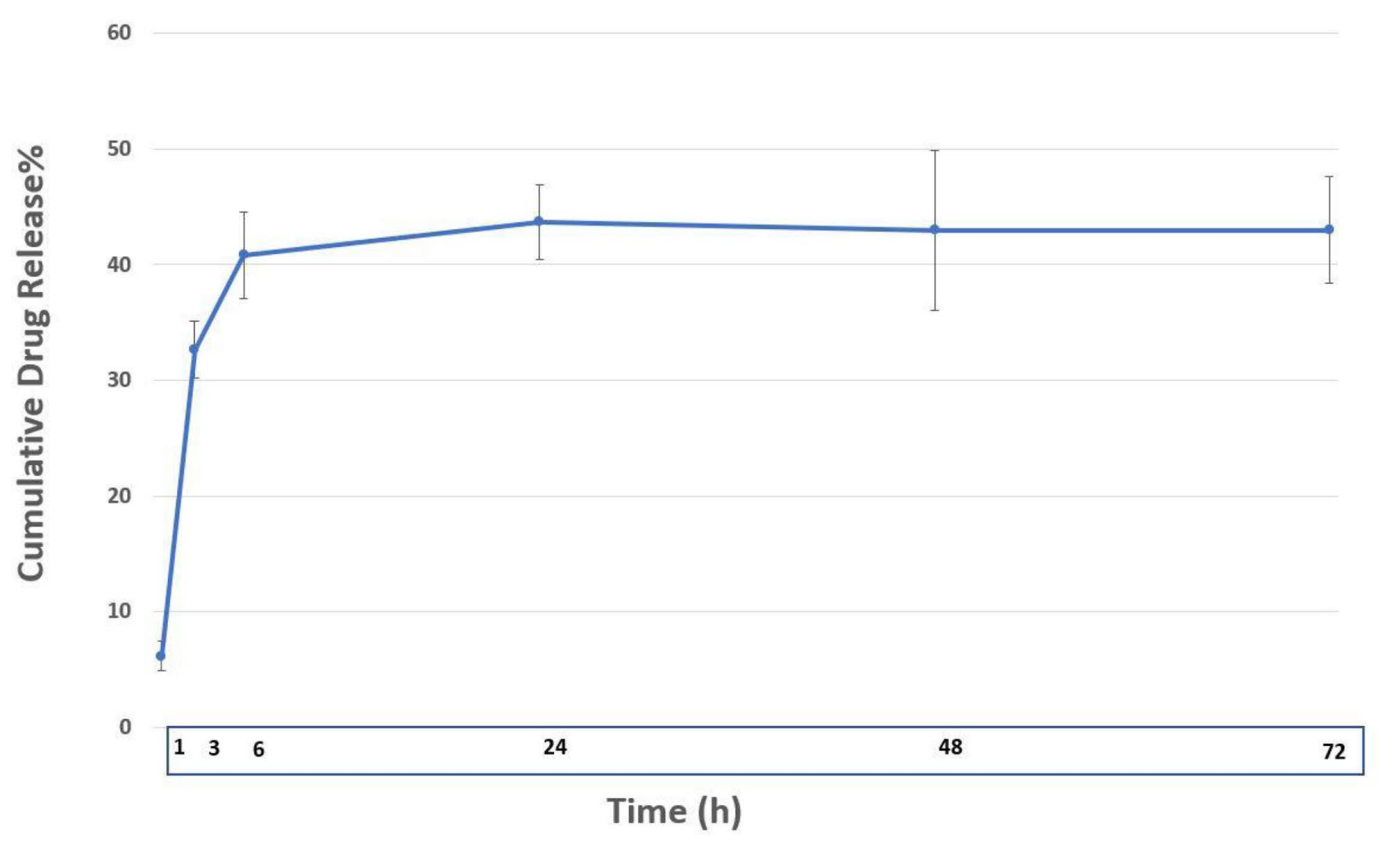
Cumulative release profile of cinnamon oil from CO-SLN in 72 h

The amounts of drug release of the formulation are showed in Fig. 3. Maximum cumulative release reached to amount of 44.14% in 72 h which reveals a sustain controlled release of CO for long term, however, an initial burst release of 53.9% in 6 h was observed. The highest regression coefficient (R^2^) among zero order, first order, Higuchi, Korsemeyer-Peppas and Hixson crowell models was considered as the best fitted kinetic model for the formulation. Drug release kinetic in SLNs, showed the highest regression coefficient with Hixson crowell model with R^2^ = 0.9956 (Table 1).

**Table 1:** Release kinetic parameters for CO-SLN based on different mathematical models

formulation	Zero order		First order		Higuchi		Korsemeyer-peppas		Hixson–Crowell	
	K_0_	R^2^	K_1_	R^2^	K_H_	R^2^	K_K_	R^2^	K_Hc_	R^2^
**CO-SLN**	14.375	0.836	-0.092	0.777	14.375	0.836	0.446	0.988	0.026	0.996

### Cell viability

**Fig. 4:**
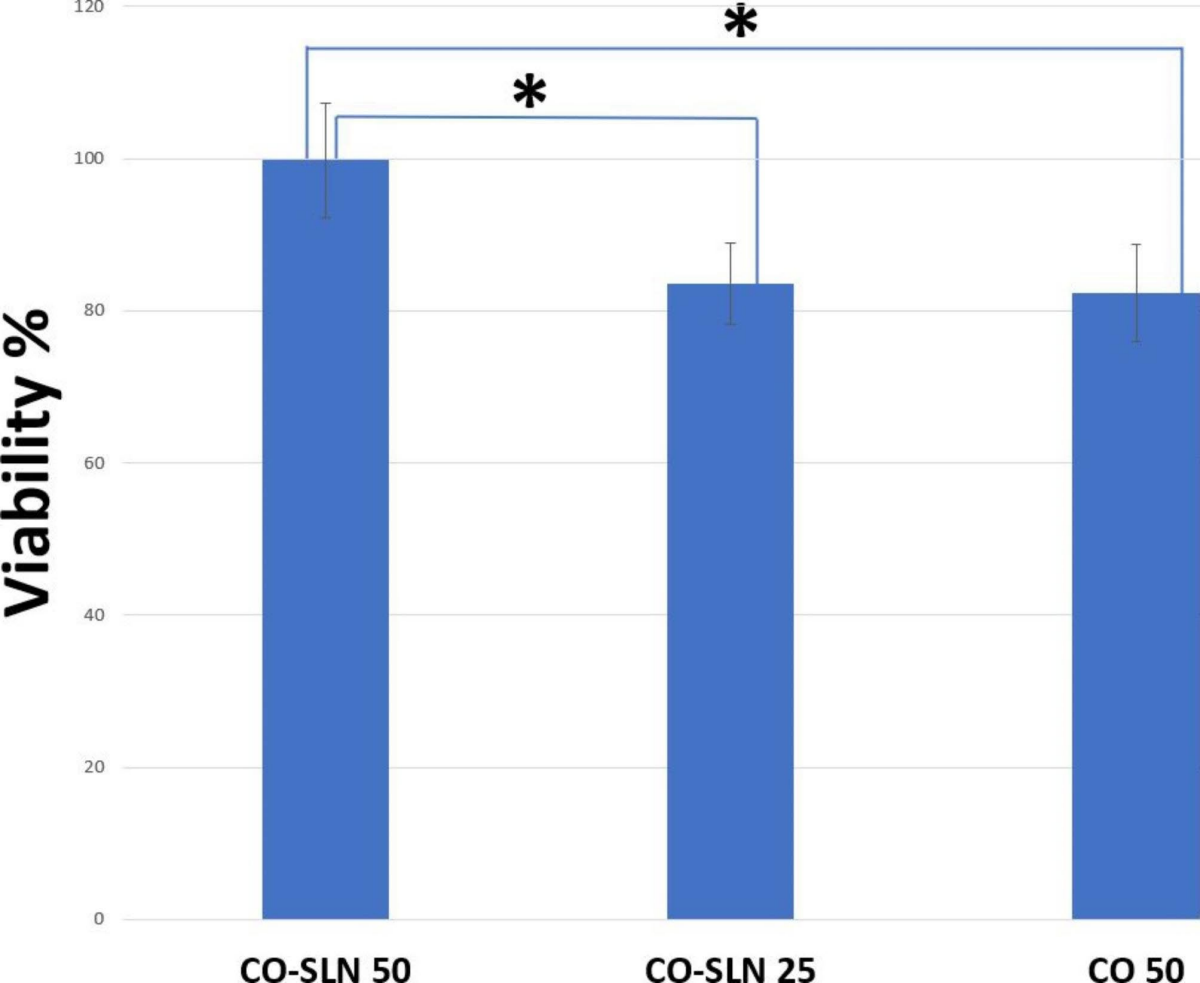
The cell viability percent in contact with CO (50 mg/ml) in comparison with CO-SLN (25, 50 mg/ml)

Probable toxicity of the CO and CO-SLN was initially determined on HU02 (Foreskin fibroblast) cell lines using an MTT method. The CO concentration was 50 mg/ml while CO-SLN was evaluated with the concentrations of 25 and 50 mg/ml. Results revealed that the cell viability with CO-SLN (50 mg/ml) was 99.8% which was significantly higher than CO-SLN (25 mg/ml) (83.6%) and CO (82.3%) groups (Fig. 4).

### Antibacterial activity

#### Antibiotic susceptibilities against *E. coli* isolates

Strains isolated from the hospital were examined using disk diffusion method and 10 strains with highest antibiotic resistance were selected for further evaluations. The results of the antibiogram test of eight antibiotics against MDR *E. coli* are presented in Table 2.

**Table 2:** The antibiogram test of eight antibiotics against selected pathogenic MDR E.coli isolates

isolates	Ampicillin	Gentamicin	Amikacin	Ceftazidime	Cefepime	Ceftriaxone	Ciprofloxacin	Trimethoprim-Sulfamethoxazole
1	R	S	I	I	I	R	S	R
2	R	S	S	R	R	R	S	R
3	R	R	R	R	R	R	S	R
4	R	R	R	R	R	R	R	R
5	R	R	R	R	R	R	S	R
6	R	R	R	R	S	R	R	R
7	R	R	R	R	R	R	R	R
8	R	R	R	R	S	R	R	R
9	R	R	S	R	R	R	S	R
10	R	R	S	R	R	R	R	R

Breakpoints were defined for designating isolates as antibiotic susceptible (S), intermediate (I), and resistant (R)

#### Determination of the MICs and MBCs of CO-SLN and CO

The MIC and MBC of CO-SLN and CO using micro-broth dilution method were equal to 65 µg/ml, 230 µg/ml and 160 µg/ml, 550 µg/ml against the standard sample of *E. coli* (ATCC 25,922), respectively (Table 3a). Next, the MIC values ​​of all 10 MDR *E. coli* isolates were determined incorporating different concentrations of CO-SLN and CO. Results are summarized in Table 3b. As shown, the CO-SLN revealed MIC values of 60–75 µg/ml and CO showed the antibacterial activity with MICs of 155–165 µg/ml. These results confirmed the significant antibacterial activity of CO-SLN against MDR strains isolated from the hospital. The MBC results for all strains are also presented in Table 3b. The CO-SLN and CO had the MBC values ​​of 220–235 µg/ml and 540–560 µg/ml, respectively. Comparing the MIC and MBC values between CO-SLN and CO shows the significant stronger antibacterial activity of CO-SLN against MDR *E.coli* strains (P-value < 0.05).

**Table 3:** **a**. The MICs and MBCs of Nanoparticles and pure oil against E. coli (ATCC 25,922)

	Nanoparticles			Pure oil	
	MIC (µg/ml)	MBC (µg/ml)		MIC (µg/ml)	MBC (µg/ml)
E. coli (ATCC (25,922)(	65	230		160	550

**Table 3:** **b.** The MICs and MBCs value (µg/mL) for Nanoparticles and pure oil against MDR E.coli isolates

	Nanoparticles			Pure oil	
Isolates	MIC	MBC		MIC	MBC
1	70	235		160	550
2	70	230		160	550
3	65	230		155	540
4	70	230		160	550
5	60	220		160	540
6	60	220		155	550
7	70	235		160	550
8	75	235		165	560
9	70	230		160	550
10	65	230		160	550

#### Anti-biofilm activity

**Fig. 5:**
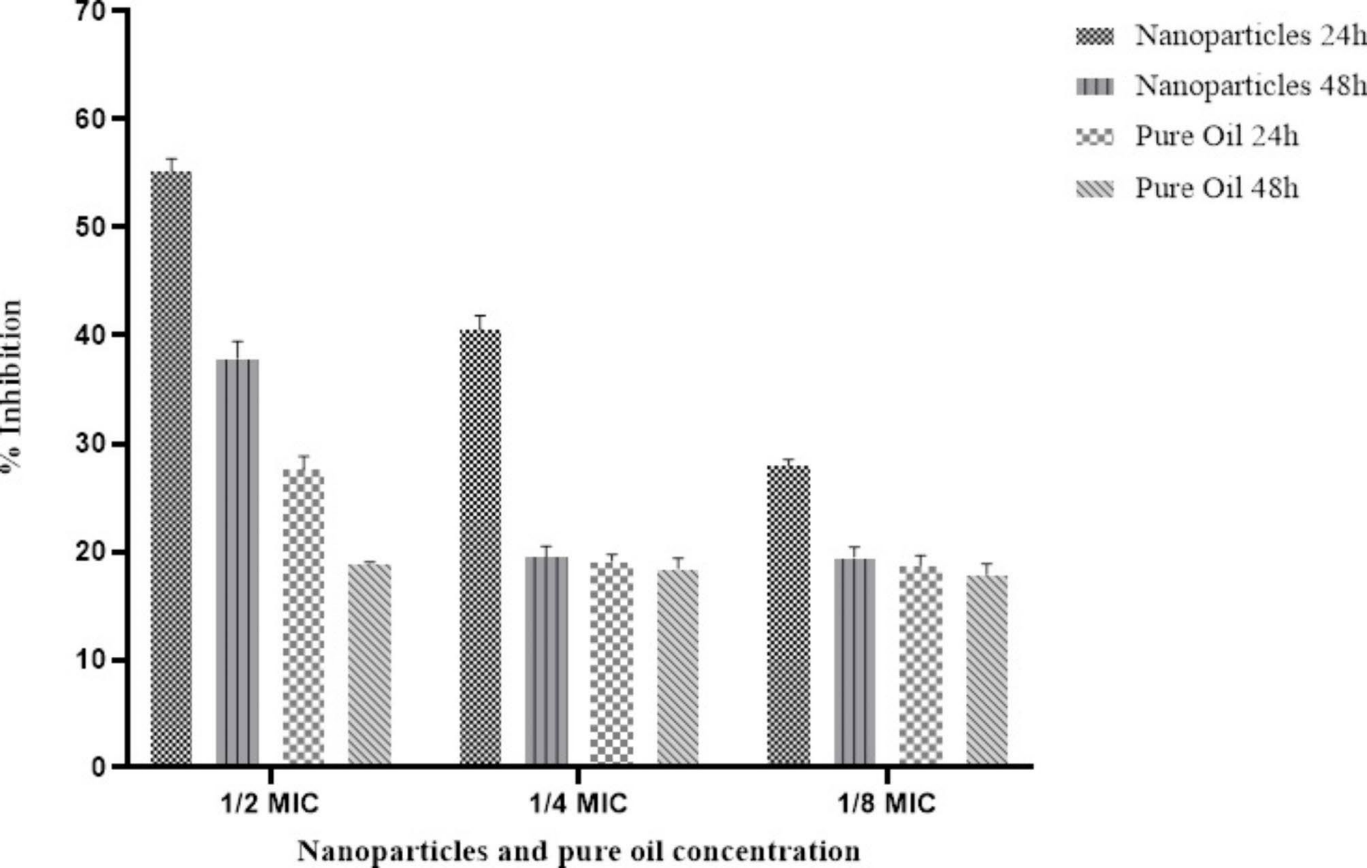
Anti-biofilm activity of the Nanoparticles and pure oil against *E. coli* isolate 4

Based on the initial antibacterial results, strain number 4 with the highest antibiotic resistance was selected for evaluation of antibiofilm activity. According the Fig. 5, CO-SLN with 1/2 MIC concentration showed the highest anti-biofilm activity (55.25%) after 24 h of incubation; and at concentrations of 1/4 and 1/8 MIC the biofilm inhibition rate was 40.54% and 27.95%, respectively. Also, the inhibition rate of CO at 1/2 MIC concentration was 27.44%, after 24 h of incubation which was a significantly lower rate in comparison with CO-SLN (p < 0.0001). Worth mentioning, the anti-biofilm activity of CO-SLN and CO after 48 h of incubation at concentrations of 1/4 and 1/8 MIC was not significantly different.

## Discussion

Recent decades were along with serious investigations for discovering new and more effective antibacterial agents, especially against MDR microorganisms [36]. Among them, natural-based materials have gained great focus due to inherent antimicrobial effects and the long history of administrations in traditional medicines as well as probable different mechanisms of action with conventional antibiotics in some herbal sources [37–39]. Cinnamon is one of the sources which has been widely investigated against microorganisms, in oil or extract form, separately or in combination with other antimicrobial agents [40, 41].

This study evaluated the antibacterial and anti-biofilm activity of CO-SLN and pure CO against MDR *E. coli* which were identified in hospital infections. Since *E.coli* is one of the susceptible microorganisms in contamination of pharmaceutical and food products, especially oral dosage forms, an effective antimicrobial agent against *E.coli* could be considered as a potentially natural preservative [42].

However, cinnamaldehyde as the major constituent of CO (responsible for its antibacterial characteristics), is a FDA approved phytoactive molecule with biocompatibility and low toxicity, it has been reported occasionally to show hypersensitivity and oral adverse reactions [43]. Hence, incorporation of natural excipients e.g., cholesterol and lecithin for fabrication of SLN drug carrier in order to lessen the required effective dose (MIC and MBC) and increase in biocompatibility was considered.

Several studies have evaluated the antimicrobial characteristic of CO in bulk form [11]. Recently the influence of incorporating nano-delivery systems on antimicrobial activity of cinnamon oil has been of interest. Among them nanoemulsions [44, 45], nanosponges [46], polymeric nanoparticles [28, 29] and lipid nanoparticles (NLC) are mainly investigated. Nevertheless, SLN as a lipidic nanoparticle has not yet been addressed as a potential delivery system for CO to enhance its antibacterial properties.

Obtained MIC for pure CO in our study was 155–165 µg/ml which is in a similar range with the study of Lei, et al. (MIC of CO against *E.coli*: 100–400 µg/ml ) [47]. However, there are other studies reported the MIC of CO, in a significantly higher ranges: (1000 µg/ml ) [48], (625–2500 µg/ml ) [49] while, El Atki, et al. reported a much lower MIC (4.88 µg/ml) [50] of CO against *E.coli*. Comparing the MIC of pure CO and CO-SLN in our results reveals that MIC for CO-SLN significantly decreased (60–75 µg/ml) (P-value < 0.05). Since one of the discovered cinnamon’s antimicrobial mechanisms of action is a destabilization of the cytoplasmic membrane [39, 51], it seems that natural lipids constructing the SLN in this study, facilitate and promote the integration and transportation of the CO through cell membrane, leading to a decrease in required doses. Additional studies have designed nano-delivery systems for CO following the goals of counteracting its high volatility, odor, rapid decomposition, poor bioavailability and prolonging its biocidal efficacy in which our results are also confirmed; for example, Bravo Cadena et al. encapsulated cinnamaldehyde into mesoporous silica nanoparticles which revealed an anti-*Pseudomonas syringae pv*. *pisi* activity with up to 90,000-fold lower concentration than concentrations of free cinnamon oil [52]. A similar finding was observed in other essential oils such as anise oil on *Listeria monocytogenes* and *E. coli* [53] and peppermint oil on *L. monocytogenes* and *S. aureus* [54]. However, Radi et al. reported that the MIC values for CO loaded NLC (nanostructured lipid carriers) were more than two folds higher than the CO (0.425 vs. 1 mg/mL) against *P. citrinum* and *P. expansum*; therefore, they hypothesized that CO is strongly entrapped in NLC and they take the advantage of its sustained and long term release [55].

The anti-biofilm activity of CO was also investigated in our study. In this study, the highest activity was related to CO-SLN with ½ MIC concentration in 24 h (55.25%) while the CO activity was 27.44%, confirming the higher antimicrobial potential of SLN delivery system, determined in MIC evaluation. Chengrong Lu, et al. assessed the anti-biofilm activity of cinnamon ethanolic extract on *E.Coli* in which the inhibition rate was 48.18% at MIC concentration [56]. It seems that however the inhibition rate was higher in cinnamon extract in comparison with CO, it may be due to higher concentration (2-fold higher). Likewise, other researches evaluated the anti-biofilm activity of Cinnamon oil or extract [57], nanoemulsion [41] or its major constituents such as cinnamaldehyde [58].

## Conclusion

In this study, we developed a two step simple method for the fabrication of CO-SLN using natural based lipids (lecithin and cholesterol) in order to increase the anti-microbial effects and decrease the required effective dose of CO against MDR *E. coli*. Results revealed that CO-SLN possesses appropriate Physico-chemical characteristics including the size (337.6 nm) with zeta potential of -26.6 mV, with spherical shape and smooth morphology. Higher cell compatibility was observed in CO-SLN in comparison with pure CO in MTT assay and MIC and MBC of CO against *E. coli*. (ATCC 25,922) decreased from 160 µg/ml and 550 µg/ml to 65 µg/ml and 230 µg/ml for CO-SLN, respectively. This result was similarly repeated for MDR *E. coli*. Generally, the discussed method can be employed for the entrapment of other natural oils for the development of more effective and less toxic, and long-lasting antimicrobial systems for various industrial and biomedical demands.

## Electronic supplementary material

Below is the link to the electronic supplementary material.

Supplementary Material 1

## Data Availability

All datasets generated for this study are included in this published article.
