# Supplementary material for: Formulation of Cinnamon (Cinnamomum verum) oil loaded solid lipid nanoparticles and evaluation of its antibacterial activity against Multi-drug Resistant Escherichia coli
Source: BMC Complement Med Ther. 2022 Nov 9;22:289. doi: 10.1186/s12906-022-03775-y (PMC9647953; doi:10.1186/s12906-022-03775-y)
Supplement: Supplementary file 1 — Supplementary Material 1 [file 12906_2022_3775_MOESM1_ESM.docx]

Supplementary data 1:


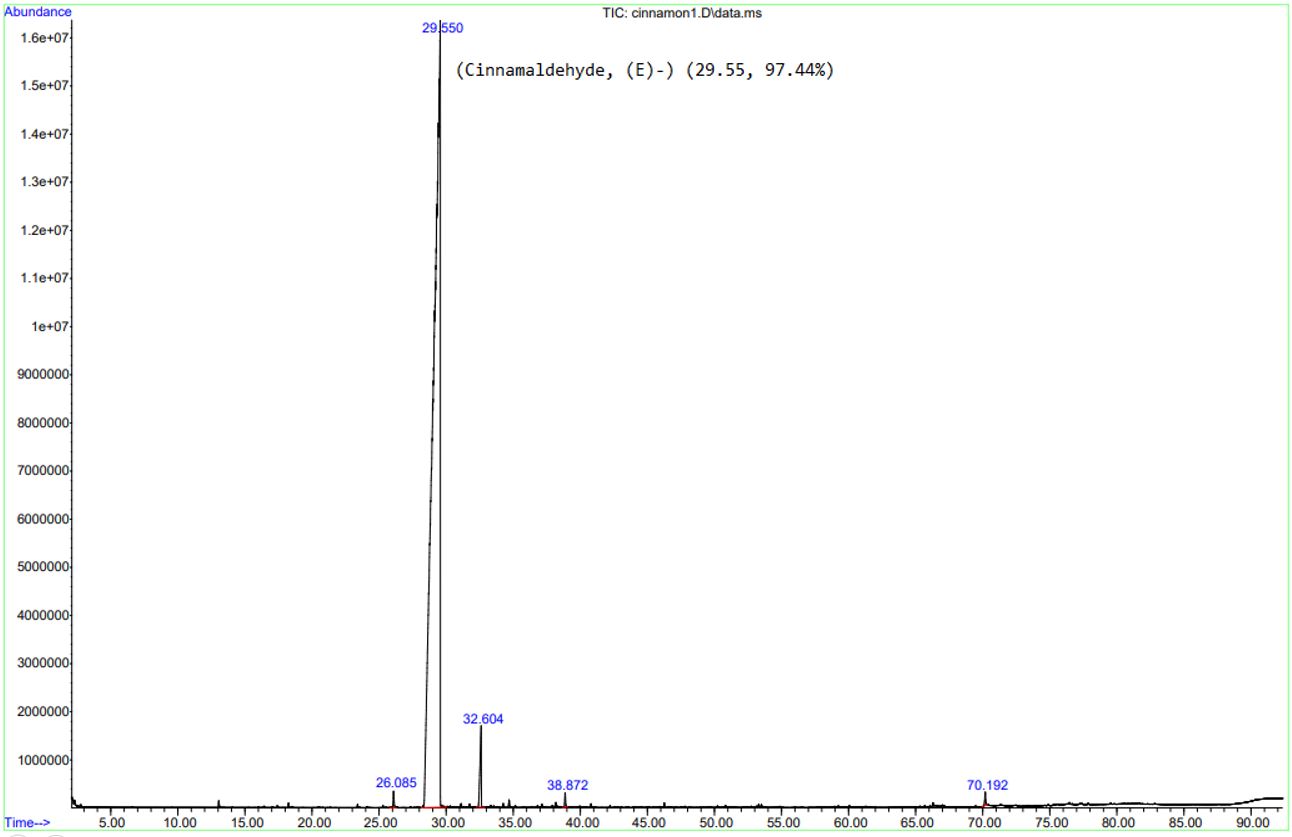


S_1_. GC-MS analysis of cinnamon oil, revealing a high concentration of cinnamaldehyde as major constituent.
